# Catalytic Dehydration of Fructose to 5-Hydroxymethylfurfural (HMF) in Low-Boiling Solvent Hexafluoroisopropanol (HFIP)

**DOI:** 10.3390/molecules23081866

**Published:** 2018-07-26

**Authors:** Sarah Tschirner, Eric Weingart, Linda Teevs, Ulf Prüße

**Affiliations:** Thünen Institute of Agricultural Technology, Bundesallee 47, 38116 Braunschweig, Germany; sarah.tschirner@thuenen.de (S.T.); eric.weingart@thuenen.de (E.W.); linda.teevs@thuenen.de (L.T.)

**Keywords:** HMF, HFIP, fructose, ion-exchange resin, Lewatit K2420

## Abstract

A mixture of hexafluoroisopropanol (HFIP) and water was used as a new and unknown monophasic reaction solvent for fructose dehydration in order to produce HMF. HFIP is a low-boiling fluorous alcohol (b.p. 58 °C). Hence, HFIP can be recovered cost efficiently by distillation. Different ion-exchange resins were screened for the HFIP/water system in batch experiments. The best results were obtained for acidic macroporous ion-exchange resins, and high HMF yields up to 70% were achieved. The effects of various reaction conditions like initial fructose concentration, catalyst concentration, water content in HFIP, temperature and influence of the catalyst particle size were evaluated. Up to 76% HMF yield was attained at optimized reaction conditions for high initial fructose concentration of 0.5 M (90 g/L). The ion-exchange resin can simply be recovered by filtration and reused several times. This reaction system with HFIP/water as solvent and the ion-exchange resin Lewatit K2420 as catalyst shows excellent performance for HMF synthesis.

## 1. Introduction

**Scheme 1:**
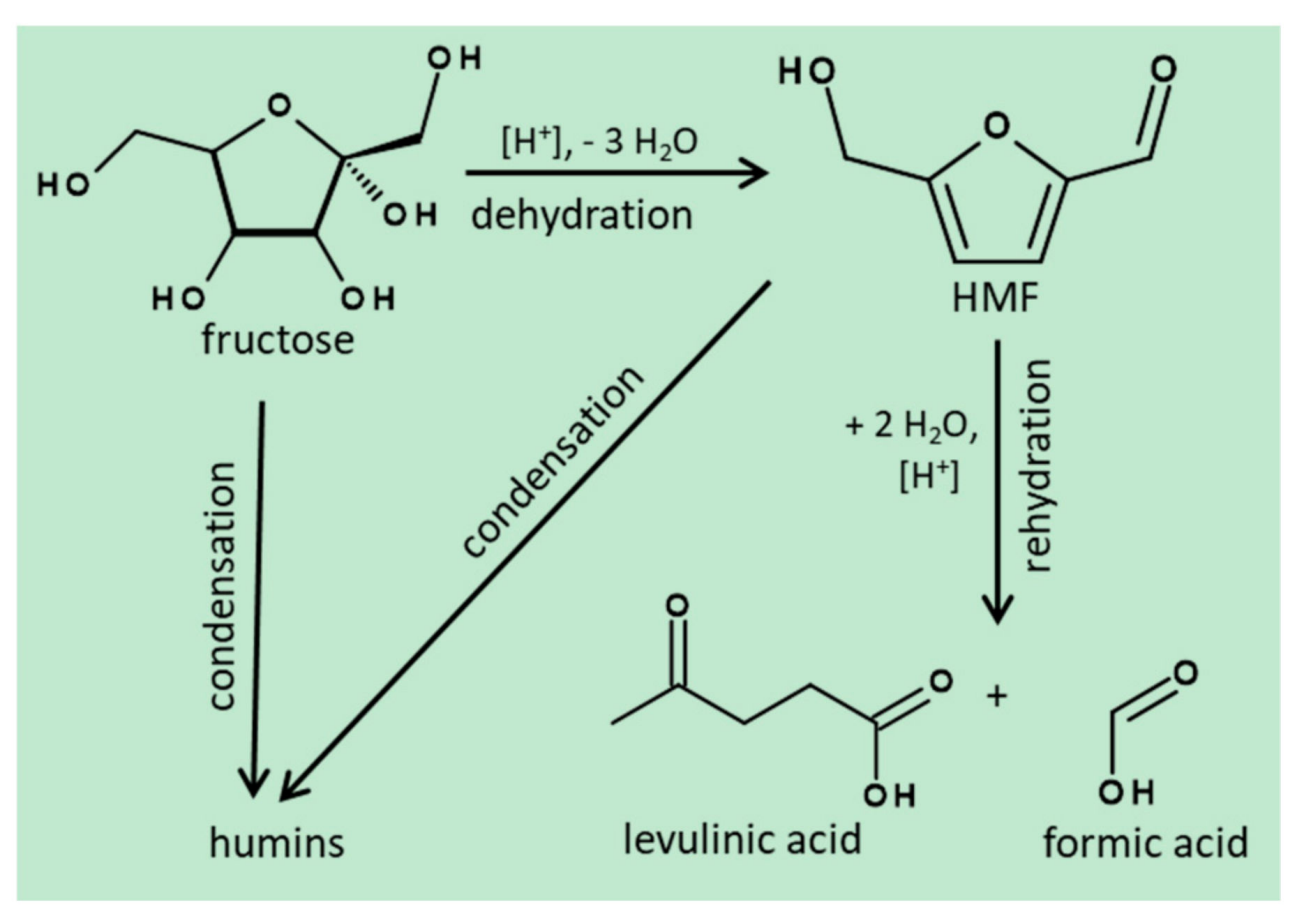
Reaction pathway from fructose to 5-hydroxymethylfurfural (HMF) and its decomposition.

Diminishing fossil resources, growing world population and increasing importance of global warming make it necessary to find alternative sources for energy and chemicals. Biomass offers a promising alternative for a sustainable supply of energy and valuable chemicals. One of the most interesting organic compounds that can be obtained from biomass is 5-hydroxymethylfurfural (HMF). Because of its functionalities, HMF is a very important key intermediate with a wide variety of products, such as furan-based polymers, fuels, fine chemicals and pharmaceuticals [1,2,3,4]. One product with great potential is furandicarboxylic acid (FDCA). Due to the structural similarity to terephthalic acid (TA), FDCA serves as possible alternative in the production of plastics. In combination with ethylene glycol it can be polymerized to bio-based poly(ethylene-2,5-furanoate) (PEF), an alternative to the petrochemical-based polyethylene terephthalate (PET). The PET market volume of 50 million t/a demonstrates the high potential of FDCA and the importance of HMF production. Therefore, in the last 30 years the dehydration of fructose to HMF, which is the most efficient synthetic route, received increasing attention. The main problem in the production of HMF is the instability of HMF. In the presence of water and acid the formed HMF is rehydrated and different condensation products are formed. As a result, levulinic and formic acid in a subsequent rehydration reaction, as well as soluble and insoluble polymers (humins) in parallel condensation reactions, are generated (Scheme 1).

**Scheme 2:**
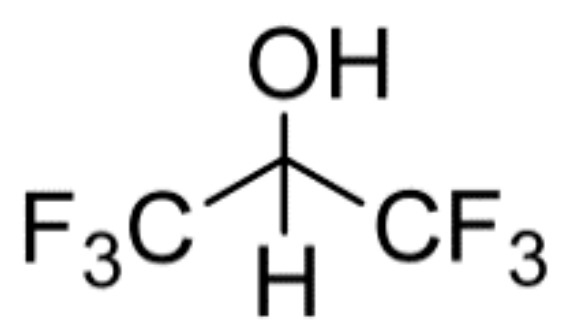
Structure of 1,1,1,6,6,6-hexafluoroisopropanol (HFIP).

To overcome HMF condensation and rehydration reactions, different approaches such as the use of biphasic reaction systems or organic solvents are investigated. In biphasic systems, the reaction takes place in an acidic aqueous phase and the formed HMF is extracted in situ into the organic phase. Typically applied extracting solvents are methyl isobutyl ketone (MIBK), 2-butanol or mixtures of different solvents. With a 2-butanol–water biphasic system, HMF yields up to 89% were observed [5,6]. Yang et al. achieved the HMF yield of 89% with the biphasic mixture of water and 2-butanol (2:3 *v*/*v*) for the dehydration of fructose and the use of hydrated niobium pentoxide as catalyst [5]. With a combination of MIBK and 2-butanol as extraction solvent, HMF yields of 69% up to 82% were observed [7,8]. The HMF yield of 69% with a fructose conversion of 86% was obtained with the biphasic solvent system water–MIBK–2-butanol and HCl as catalyst [7]. The replacement of a part of the water phase by dimethyl sulfoxide (DMSO) with MIBK and 2-butanol as extraction solvents led to an increase of the HMF yield to 82% with a fructose conversion of 95% [8]. However, small partition coefficients for HMF of 1.6–1.9 [7], high amounts of solvent and high boiling points of the solvents (b.p. (MIBK) = 116 °C, b.p. (2-butanol) = 100 °C) lead to difficulties in HMF production and isolation. In previous investigations we showed that hexafluoroisopropanol (HFIP, Scheme 2) solves these problems in an excellent way [9,10].

In biphasic systems, HFIP showed a very good extraction capacity and an extraordinarily high partition coefficient for HMF of >10. In combination with the low boiling point of 58 °C it is a superior extraction solvent for the HMF production.

Good results in the dehydration of fructose to HMF are also achieved with the use of monophasic reaction systems. Organic solvents, like dimethyl sulfoxide (DMSO), dimethylformamide (DMF) and 1,4-dioxane or ionic liquids were applied [11,12,13,14]. Shimizu et al. reported for DMSO as reaction solvent and the acidic ion-exchange resin Amberlyst 15 as catalyst an HMF yield of 92% at 120 °C under reduced pressure and 2 h reaction time [11]. For fructose dehydration, either in DMSO or in [BMIM]Cl, high HMF yields of around 95% were described at 120 °C with a chromium-exchanged K-10 clay as catalyst [15]. The high boiling point or no boiling point of these solvents leads to the problem of isolation of the thermally labile HMF. With the use of low-boiling HFIP in combination with water as monophasic reaction solvent instead, a very good result was achieved. After 360 min for 0.1 M fructose, 12.5 vol % water in HFIP, 18 g/L Amberlyst 15 and 87 °C, an HMF selectivity of 79% at a fructose conversion of 98% was received [9]. Based on this result, further investigations on the influence of different reaction parameters on fructose dehydration were carried out. In this paper we present a monophasic reaction system with HFIP and water as reaction solvent and strong acidic ion-exchange resins as catalysts. Excellent performance in view of HMF synthesis from fructose was observed. An HMF yield up to 76% at high initial fructose concentrations of 0.5 M can be achieved at optimized reaction conditions, and the recyclability of the catalyst was proven.

## 2. Results and Discussion

### 2.1. Screening of Different Ion-Exchange Resins for Dehydration of Fructose

**Figure 1:**
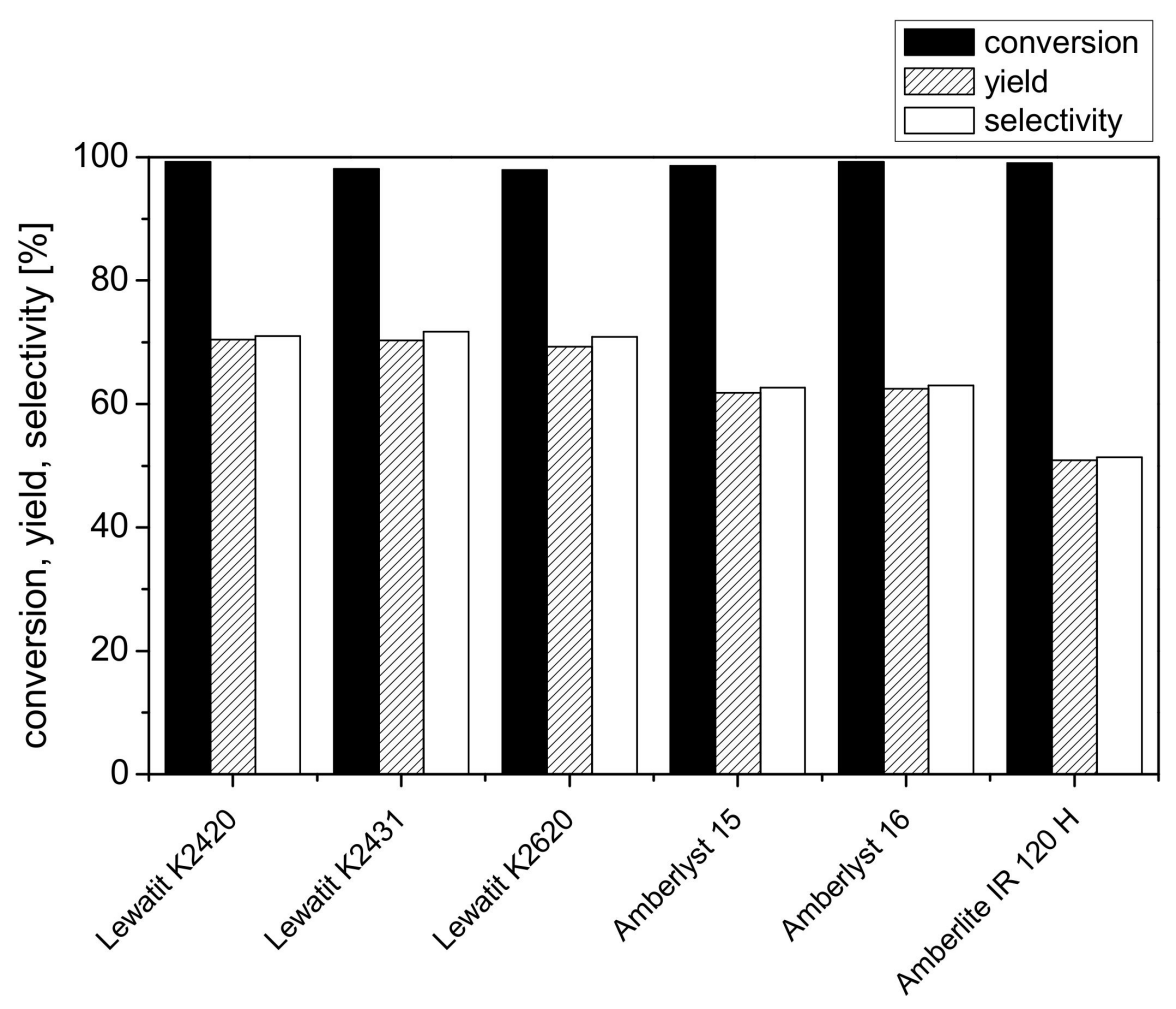
Screening of different ion-exchange resins and their influence on fructose conversion, maximum HMF yield and maximum HMF selectivity. Reaction conditions: 0.5 M fructose, 12.5 vol % water in HFIP, 20 g/L (21.6 mol % (acid sites/substrate)) catalyst and 105 °C.

Cation-exchange resins are known as suitable solid acid catalysts for HMF synthesis [1,16]. Six different commercially available acidic ion-exchange resins (properties of the used cationic-exchange resins are summarized in Table S1) were tested for the catalysis of fructose dehydration. Most of the chosen catalysts are strongly acidic macroporous polystyrene- and divinylbenzene copolymer-based ion-exchange resins, except for Amberlite IR-120 (H), which is a microporous gel-type strong acid ion-exchange resin. Gel-type resins have smaller pore diameters than macroporous resins. The copolymer matrix of polystyrene and divinylbenzene of the macroporous resins are cross-linked in comparison to gel-type resins, leading to different pore systems with varying pore diameters and volumes (Table S1). The effect of the different catalysts on fructose conversion, maximum HMF yield and maximum HMF selectivity was investigated and is shown in Figure 1.

Under screening reaction conditions, the highest HMF yields in the range of 69–70% were obtained for Lewatit-type resins. With Amberlyst resins, an HMF yield of 62% was achieved. The lowest HMF yield of 51% was reached with Amberlite IR-120 (H). Amberlite IR-120 (H) has a poor accessibility of the acid sites, due to its small pore diameters (<0.5 nm). Therefore, the macroporous ion-exchange resins obtained superior results compared to the gel-type resin, based on the better availability of the active sites for fructose [17]. Additionally, the larger acid-site concentration of Lewatit-type resins led to a faster fructose conversion. In comparison to the literature, higher HMF yields were generated with ion-exchange resins as catalyst in HFIP than in other low-boiling solvents [18,19]. Lai et al. used isopropyl alcohol as reaction solvent for HMF production. At 120 °C and with Amberlyst 15 as catalyst, a total furfural yield (including HMF, ether and acetalization products) of 60% was observed after 4 h [19]. Only in high-boiling reaction solvents like DMSO and DMF can HMF yields over 80% be achieved [11,12]. A high HMF yield was obtained with Lewatit K2420. Additionally, this catalyst provides a good thermostability up to 150 °C. Hence, Lewatit K2420 was selected as catalyst for the dehydration of fructose in the following experiments.

### 2.2. Examination of Different Reaction Parameters

The influence of various reaction parameters, like initial fructose concentration, catalyst concentration, water content in HFIP, temperature and effect of the catalyst particle size on fructose dehydration were evaluated.

#### 2.2.1. Effect of Initial Fructose Concentration

**Figure 2:**
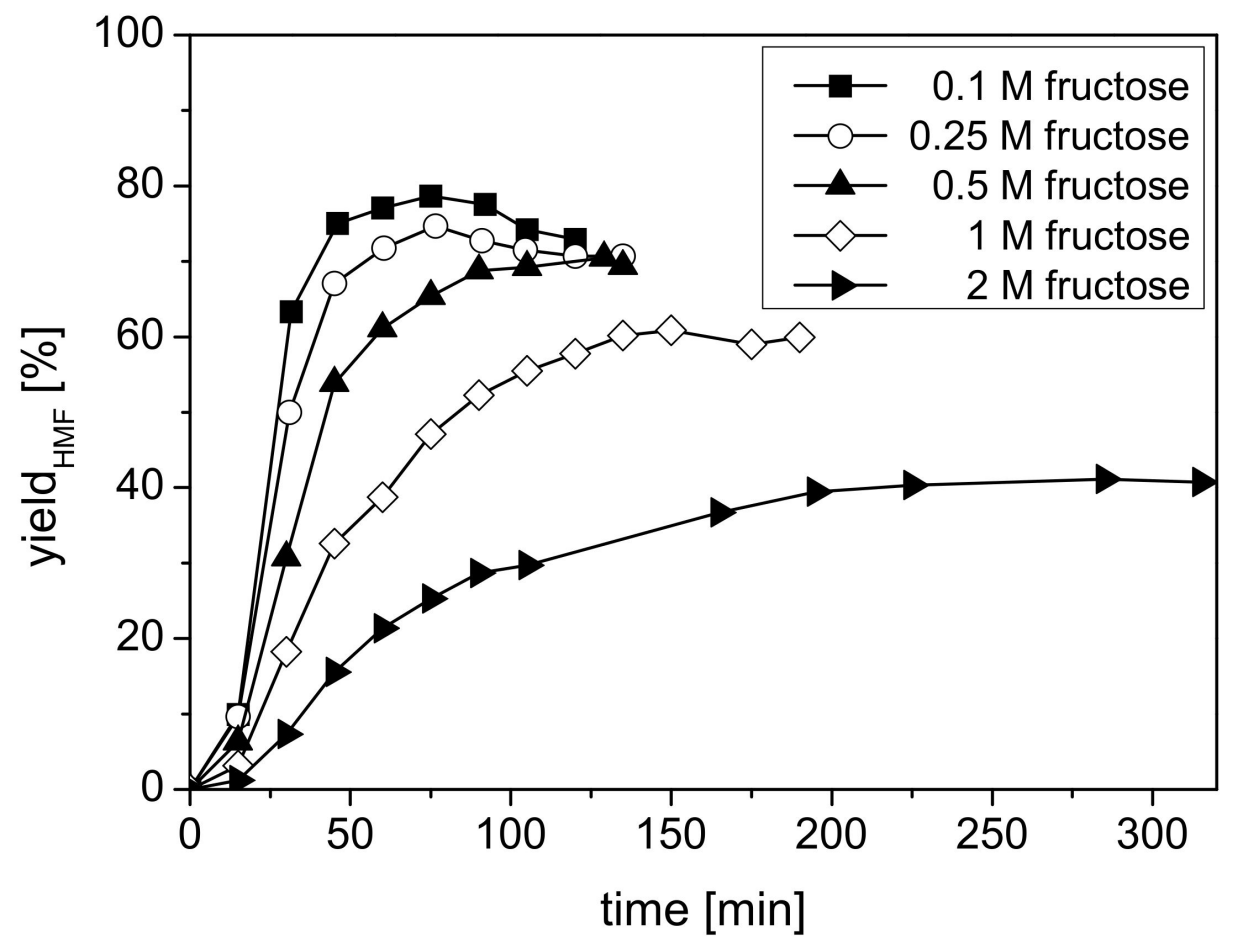
Effect of fructose concentration on the HMF yield. Reaction conditions: 0.1–2 M fructose, 15 vol % water in HFIP, 22 g/L (23.8 mol %) Lewatit K2420 and 110 °C.

High initial fructose concentrations are an important aspect in the development of an industrial HMF production process. Hence the influence of five different initial fructose concentrations on the dehydration of fructose to HMF were investigated using 22 g/L (23.8 mol %) Lewatit K2420, 15 vol % water in HFIP and a temperature of 110 °C. The fructose concentrations were varied between 0.1 M and 2 M (18–360 g/L). The effect of the initial fructose concentration on the HMF yield is shown in Figure 2.

The largest HMF yield of 79% was achieved with the lowest initial fructose concentration of 0.1 M after 75 min and a fructose conversion of 99%. The HMF yield was reduced to 75% and 70% at a fructose conversion of 99% by rising the fructose concentration to 0.25 M and 0.5 M, respectively, whilst the reaction time increased to 77 min and 129 min, respectively. Further increase of the fructose concentration led to decreased HMF yields of 61% and 41% at 99% fructose conversion for 1 M and 2 M fructose, respectively. The content of the unknown side products (humins), which was defined as amount of undetectable products by carbon balance, increased with the fructose concentration (0.1–2 M) from 7 to 37%. The summarized yields of levulinic and formic acids had also risen from 14 to 23% with increasing fructose concentration from 0.1 M to 2 M. Nevertheless, Lewatit K2420 was an effective catalyst for the fructose dehydration at high initial fructose concentrations, in comparison to the literature, which only delineates high HMF yields in high-boiling reaction solvents like DMSO and DMF at low initial concentrations [11,12]. Ohara et al. reported for the fructose dehydration in DMF an HMF yield of 90%, with a fructose concentration of 33 g/L (0.18 M) and a reaction temperature of 100 °C as well as total fructose conversion [12]. A 0.5 M (90 g/L) fructose concentration was selected for further reactions, because of the high HMF yield at large fructose concentrations.

#### 2.2.2. Effect of Catalyst Concentration

**Figure 3:**
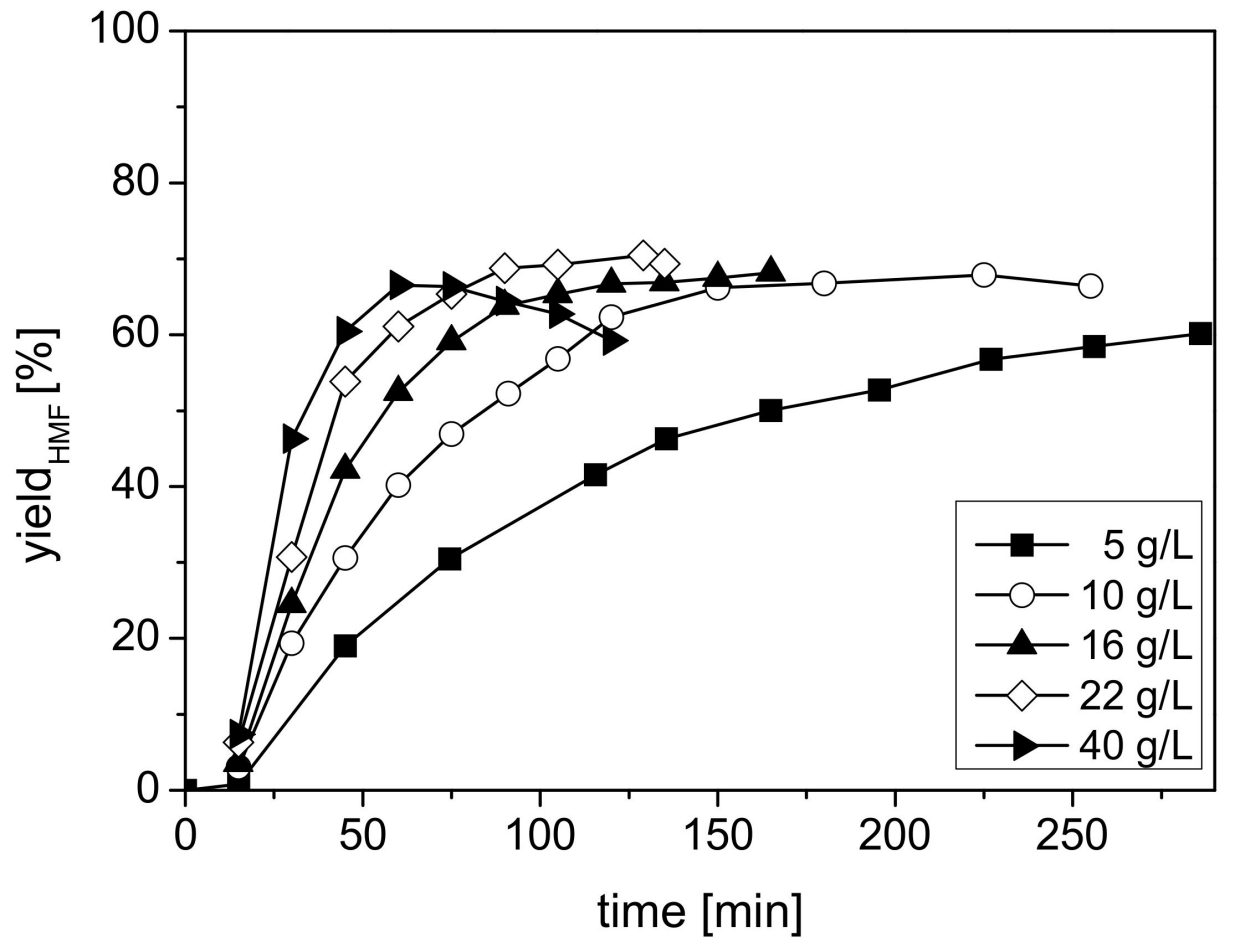
Effect of catalyst concentration on the HMF yield. Reaction conditions: 0.5 M fructose, 15 vol % water in HFIP, 5–40 g/L (5.4–43 mol %) Lewatit K2420 and 110 °C.

Figure 3 shows the effect of different catalyst concentrations (5–40 g/L; 5.4–43 mol %) on the HMF yield with equal reaction conditions.

First, the fructose dehydration was performed without catalyst. In the absence of catalyst, no HMF was formed. With increasing amount of catalyst, the HMF yield rose from 60% for 5 g/L (5.4 mol %) and 94% fructose conversion to 68–70% for a catalyst concentration in the range of 10 g/L (10.8 mol %) to 22 g/L (23.8 mol %) and 99% fructose conversion. At a higher catalyst concentration (40 g/L, 43 mol %), slightly lower HMF yield of 66% at a fructose conversion of 99% was observed. The side reactions of HMF were favored due to the larger amount of accessible acid sites, which promote the rehydration and condensation reactions to levulinic acid, formic acid and humins. A catalyst concentration of 22 g/L (23.8 mol %) was chosen as an optimal amount of catalyst for further experiments, because this concentration allowed a fast fructose conversion.

#### 2.2.3. Effect of Water Content

**Figure 4:**
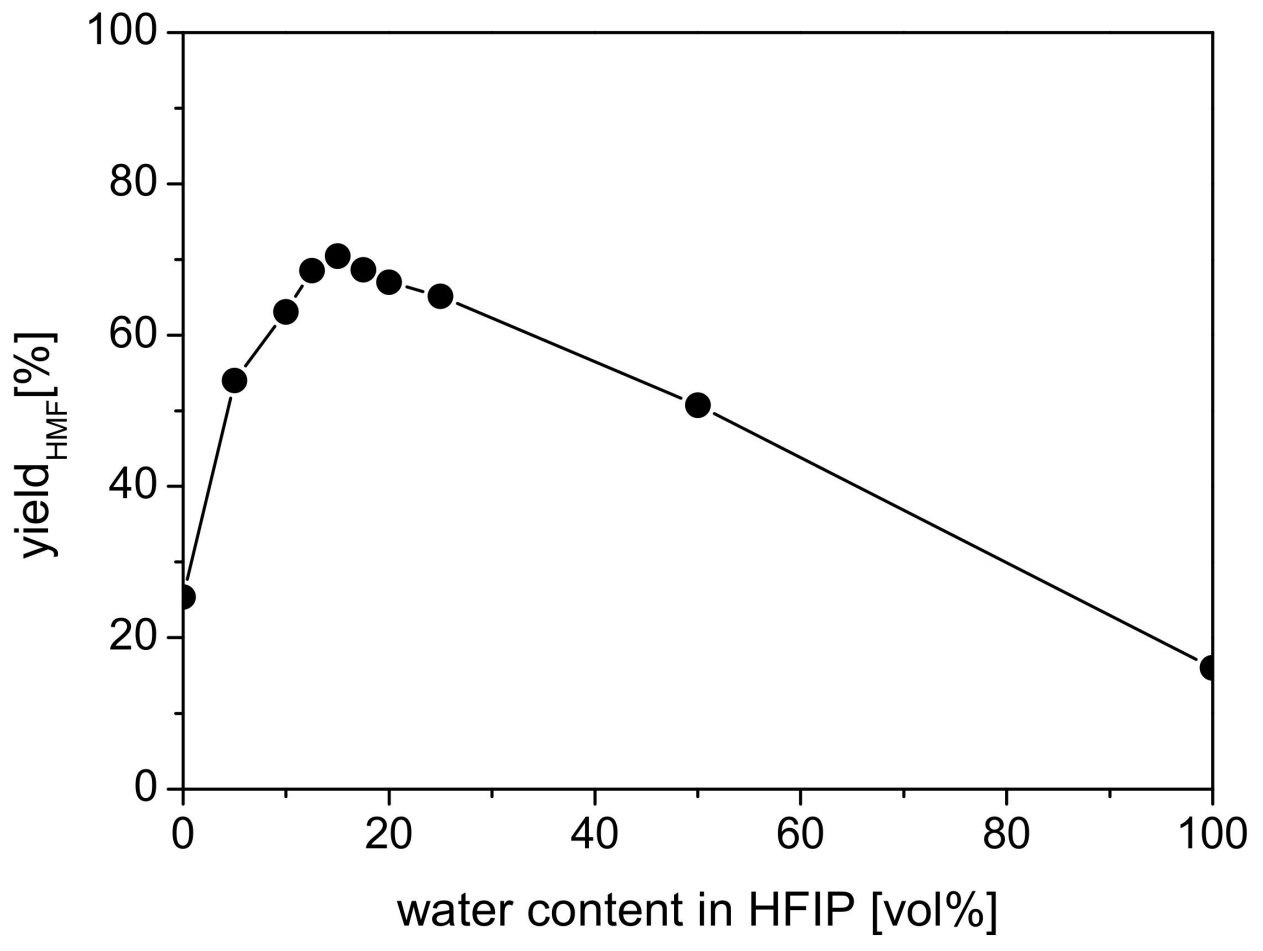
Effect of water content in HFIP on maximum HMF yield. Reaction conditions: 0.5 M fructose, 0–100 vol % water in HFIP, 22 g/L (23.8 mol %) Lewatit K2420 and 110 °C.

The addition of different amounts of water to HFIP and its effect on the maximum HMF yield was investigated. The results are illustrated in Figure 4.

The highest maximum HMF yield of 69–70% was achieved for 12.5–17.5 vol % water in HFIP as reaction solvent. With a water content <10 vol % and ≥25 vol %, the HMF yield declined significantly. An HMF yield of 25% was observed in pure HFIP solution and an HMF yield of 16% in pure water as reaction solvent. The addition of water to HFIP led to a slower conversion of fructose. With increasing water content the rehydration of HMF to levulinic acid and formic acid was favored. The summarized yield of levulinic and formic acids rose from 15 to 50% when the water content in HFIP was increased from 15 to 100 vol %. For decreasing water contents from 15 to 0 vol %, the amount of fructose condensation products (e.g., difructose anhydrides), which were not quantified, increased. H_2_O (15 vol %) in HFIP was selected as optimal water content, due to the high achieved HMF yield.

#### 2.2.4. Effect of Reaction Temperature

Seven different reaction temperatures in the range of 90 to 120 °C were examined, in order to determine their influence on fructose conversion, HMF selectivity and HMF yield. The results are shown in Table 1.

At 90 °C, an HMF yield of 64% was achieved after 405 min. With increasing temperature, the HMF yield rose to a maximum of 71% at 105 °C after 137 min. At even higher temperatures, the HMF yield dropped to 67% at 120 °C. As expected, the fructose dehydration rate increased with rising temperature. When the temperature was increased from 90 to 120 °C, the summarized yield of levulinic and formic acids rose from 7 to 23%. Consequently, the amount of unknown side products (humins) decreased with rising temperature.

A reaction temperature of 105 °C was chosen for following investigations, due to the high HMF yield and low reaction time.

#### 2.2.5. Kinetic Analysis

**Figure 5:**
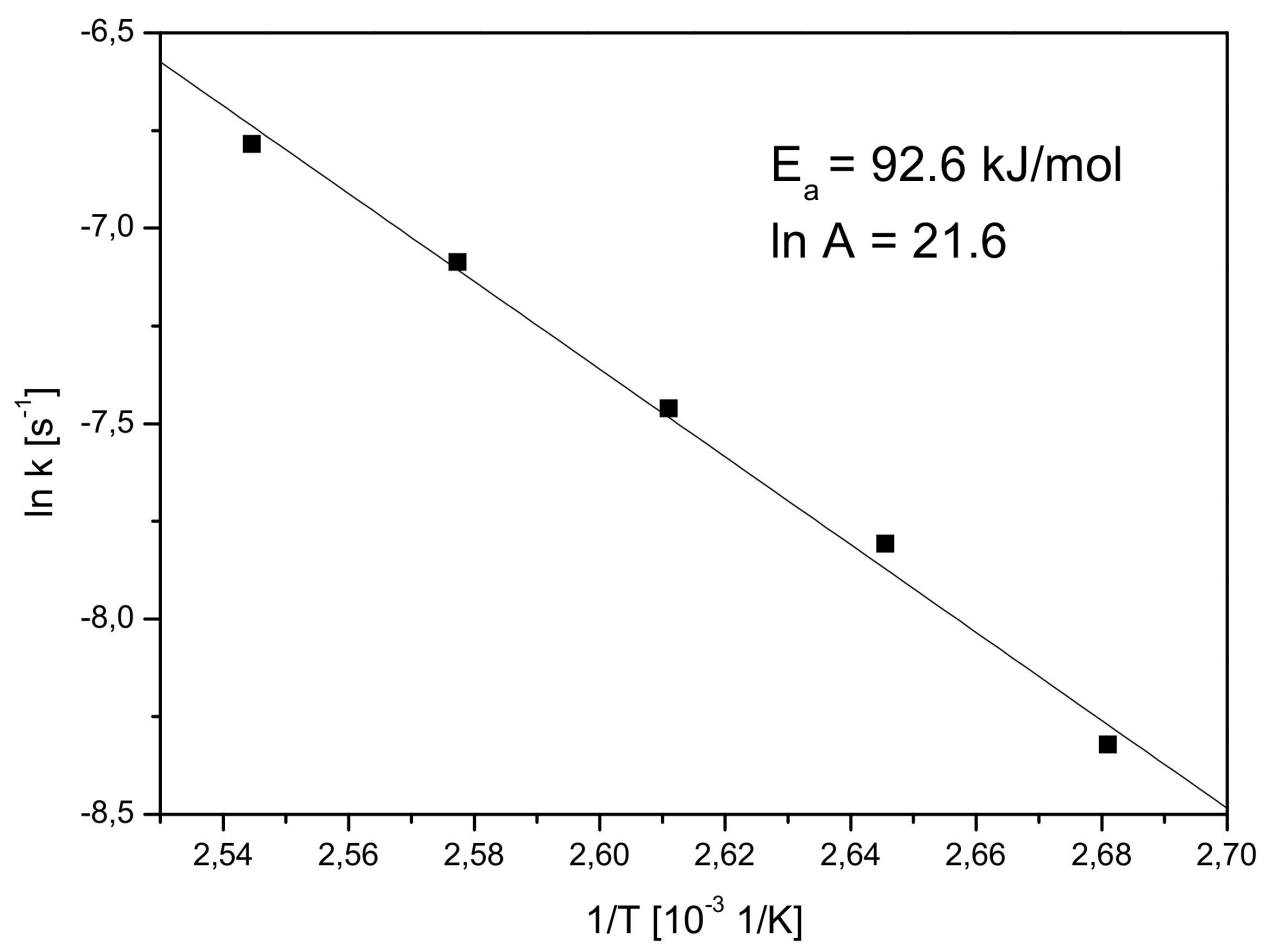
Arrhenius plot for the dehydration of fructose in 15 vol % water in HFIP, 22 g/L (23.8 mol %) Lewatit K2420 and 0.5 M fructose.

Additionally, the kinetic analysis of fructose dehydration in the HFIP/water system and Lewatit K2420 as catalyst was performed. The reaction order of one was adopted for fructose dehydration in HFIP, as described in the literature [20,21]. The logarithm of 1–X (X = conversion of fructose) was plotted versus the reaction time for each reaction temperature, and the first-order reaction constants were determined. Afterwards, the reaction constants were applied in the Arrhenius equation. The obtained Arrhenius plot is shown in Figure 5.

An activation energy of 92.6 kJ/mol was calculated, which is comparable to the ones described in previous works [14,22]. Qi et al. determined an activation energy of 103 kJ/mol for fructose dehydration in acetone–water mixture and 60.4 kJ/mol in acetone–DMSO mixture, both with addition of an acidic ion-exchange resin as catalyst [22].

#### 2.2.6. Effect of Catalyst Particle Size

The influence of the catalyst particle size on the fructose dehydration at optimized reaction conditions was investigated. The fructose conversion, HMF selectivity and HMF yield for different catalyst sizes are summarized in Table 2.

When Lewatit K2420 particles in their original size distribution were used, a fructose conversion of 98% and an HMF yield of 71% were achieved. 90% of the catalyst particles had a particle size in the range of 500–710 µm, 10% were even larger. The crushing of the ion-exchange resin particles to 150–300 µm led to a faster fructose conversion and an HMF yield of 75%. For 25–100 µm catalyst particles, an HMF yield of 76% at a fructose conversion of 99% was observed. Therefore, crushing the catalyst particles from the original size to 25–100 µm led to a faster HMF formation and a reduction of the reaction time to achieve maximum HMF yield from 137 to 120 min. A further reduction of the catalyst particle size resulted in a negligible increase of the HMF yield. In the literature, similar results were described [23]. The higher HMF yield and faster HMF formation for smaller catalyst particles can be attributed to the shorter diffusion pathway to and from acid sites of the catalyst. This results in a lower internal mass transfer limitation. The lower pore diffusion limitation is beneficial for the selective production of HMF. Since HMF is an intermediate product of the reaction, the diffusion of HMF out of the pores has to be fast, in order to avoid a subsequent rehydration reaction, which is also catalyzed by acid sites. Consequently, the amount of the rehydration products decreased with the reduction of particle size. The summarized yield of levulinic and formic acids was reduced from 15.5% at original particle size to 11.8% at particle sizes of 25–100 µm. Therefore, smaller catalyst particles in the range of 25–100 µm were used in the following experiments.

### 2.3. Recyclability of the Catalyst

**Figure 6:**
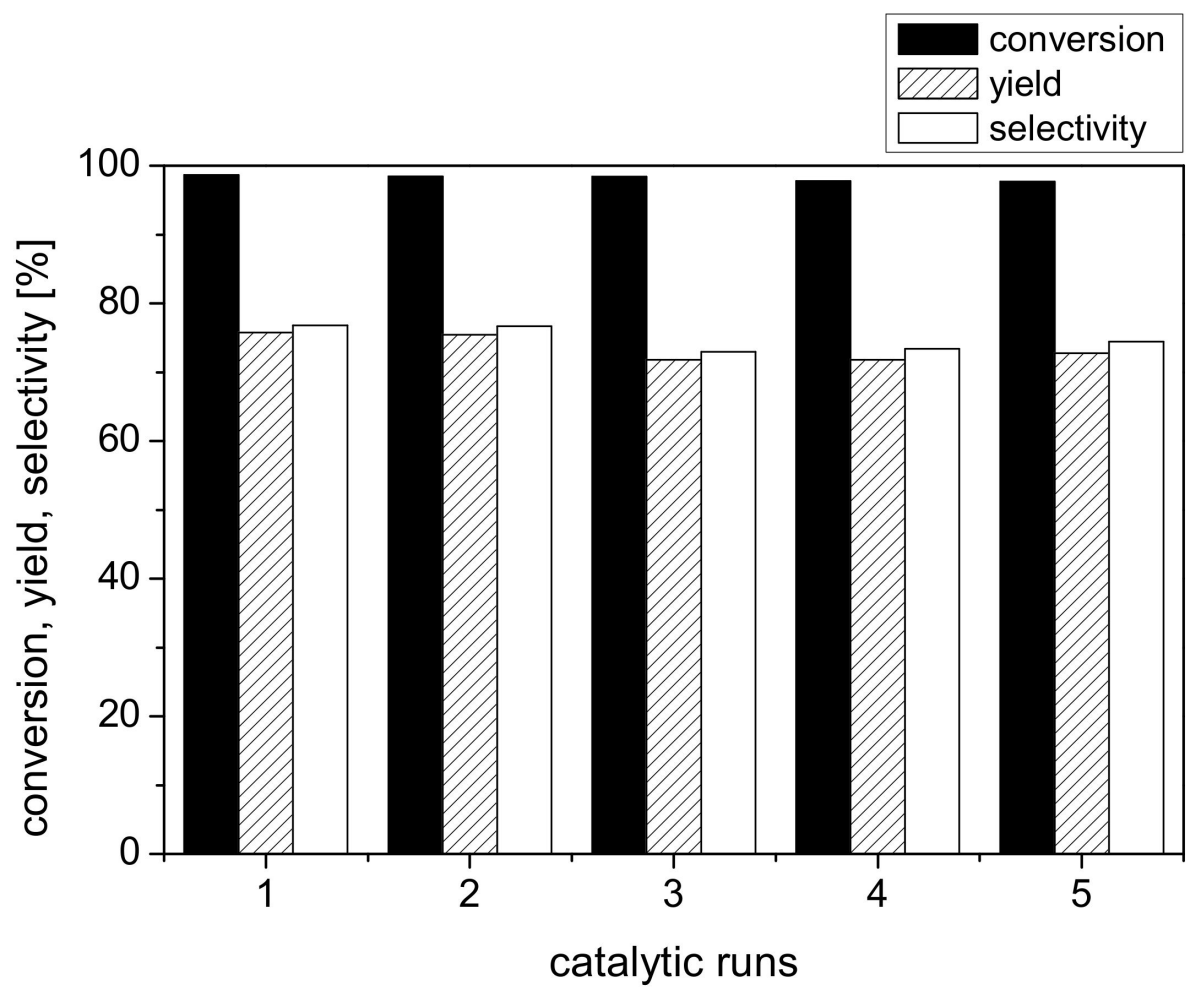
Recyclability of Lewatit K2420 (25–100 µm). Reaction conditions: 0.5 M fructose, 15 vol % water in HFIP, 22 g/L (23.8 mol %) Lewatit K2420 (25–100 µm), 105 °C and 135 min.

The reuse of the catalyst plays an important role for the development of economic processes. The recyclability test of Lewatit K2420 was performed under the previously optimized reaction conditions in five consecutive runs. Between each run, Lewatit K2420 was separated from the reaction solution by filtration and washed with HFIP. The fructose conversion, HMF yield and HMF selectivity after 135 min for each catalytic run are shown in Figure 6.

The HMF yield slightly decreased from 76 to 72%, as well as the fructose conversion from 99 to 98%. The catalytic activity showed almost constant performance over five successive runs. The fructose dehydration reaction rate dropped slightly for each catalytic run. In comparison to the literature, a very weak deactivation of the ion-exchange resin for successive runs was noticed [23,24]. In DMF as reaction solvent in combination with Amberlyst 15 as catalyst, a strong decline in HMF yield from 64 to 0% in five successive catalytic runs was observed [23]. A poor reusability in combination with a low HMF yield of 46 mol % was achieved for fructose dehydration with Amberlyst 70 in water under microwave heating. Over three reaction cycles, the HMF yield decreased by about 10% [24].

The good reusability, the high fructose concentration and the short reaction time enable high space-time yields, which are necessary for an efficient continuous HMF production. Consequently, further investigations will focus on the continuous HMF production [25].

## 3. Materials and Methods

### 3.1. Materials

Fructose (99.5% p.a. Carl Roth, Karlsruhe, Germany), HFIP (99% Fluorochem, Hadfield, UK), HMF (98%, Sigma-Aldrich, Munich, Germany), levulinic acid (99%, Merck, Darmstadt, Germany), formic acid (98% p.a., Carl Roth, Karlsruhe, Germany), Lewatit K2420 (Lanxess, Cologne, Germany), Lewatit K2430 (Lanxess, Cologne, Germany), Lewatit K2620 (Lanxess, Cologne, Germany), Amberlyst 15 (dry) (Acros Organics, Geel, Belgium), Amberlyst 16 (wet) (Fluka, Munich, Germany), Amberlite IR-120(H) (Alfa Aesar, Heyshem, UK). All chemicals were used as received. Lewatit K2420, Lewatit K2430, Lewatit K2620, Amberlyst 16 (wet) and Amberlite IR-120 (H) were washed with water (Merck Milli-Q, Darmstadt, Germany) until the permeate is colourless and dried overnight at 100 °C prior to use. HFIP was recycled by atmospheric evaporation distillation (b.p. 58 °C). No contamination in the distilled HFIP was found. HFIP was reused in subsequent reactions. Further information on HFIP is provided in Table S2 under Supplementary Materials.

### 3.2. Catalytic Conversion of Fructose into HMF

The dehydration reactions were carried out in a 1-L pressure-resistant reactor with a Teflon inlet from Parr Instrument Company. 0.5 L of the mixture of water, HFIP and fructose was filled in the reactor. Afterwards, the corresponding amount of catalyst was added to the reaction mixture and the reactor was heated to reaction temperature. The heat-up phase of the reactor was included in the reaction time. Samples were taken with a glass syringe via a septum at different times and analyzed via HPLC. For recyclability studies the catalyst was washed with HFIP, stored overnight in HFIP, filtered and reused in the following reaction. We observed little loss of catalyst (1 wt %) between each catalytic run. No fresh catalyst was added to the used catalyst.

### 3.3. Analytical Methods

Quantitative analysis was performed by HPLC (CBM-20 A, Shimadzu, Kyoto, Japan), equipped with an Animex HPX-87H column, refraction index detector (RID-6A, Shimadzu, Kyoto, Japan) and UV detector (SPD-10AV, Shimadzu, Kyoto, Japan). The reaction components were quantified by HPLC measurement at 60 °C, 0.7 mL min^−1^ flow rate of 5 mM sulfuric acid. Calibration was realized with external standards. All samples were diluted at least 10 times with water (Merck Milli-Q) prior to analysis. The fructose conversion, HMF selectivity and HMF yield were determined by Equations (1)–(3):

(1) $$\mathrm{fructose}\;\mathrm{conversion}\;\left(\text{\%}\right)=\;\frac{n_{\mathrm{fructose},\mathrm{initial}}-{\;n}_{\mathrm{fructose},\mathrm{final}}}{n_{\mathrm{fructose},\;\mathrm{initial}}}\times 100,$$

(2) $$\mathrm{HMF}\;\mathrm{selectivity}\;\left(\text{\%}\right)=\;\frac{n_{\mathrm{HMF}}}{n_{\mathrm{fructose},\mathrm{initial}}-{\;n}_{\mathrm{fructose},\mathrm{final}}}\times 100,$$

(3) $$\mathrm{HMF}\;\mathrm{yield}\;\left(\text{\%}\right)=\;\frac{n_{\mathrm{HMF}}}{n_{\mathrm{fructose},\mathrm{initial}}}\times 100.$$

## 4. Conclusions

The use of HFIP/water as reaction solvent shows excellent performance for HMF synthesis. Several heterogeneous catalysts were screened and the highest HMF yield was achieved by using the ion exchanger Lewatit K2420. The influence of different reaction conditions, like initial fructose concentration, catalyst concentration, water content in HFIP, temperature and effect of the catalyst particle size on fructose dehydration, was evaluated. At optimized reaction conditions and an initial fructose concentration of 0.5 M (90 g/L), an excellent HMF yield of 76% was achieved. Comparable results were only described for high-boiling reaction solvents, in which the isolation of thermally instable HMF is complicated. HFIP (b.p. 58 °C), on the other hand, can easily be separated from HMF by distillation. Additionally, Lewatit K2420 demonstrated a good recyclability over five repeated batch experiments.

## Figures and Tables

**Table 1:** Effect of temperature on fructose conversion, HMF selectivity and HMF yield. Reaction conditions: 0.5 M fructose, 15 vol % water in HFIP, 22 g/L (23.8 mol %) Lewatit K2420 and 90–120 °C.

Temperature (°C)	Reaction Time (min)	Fructose Conversion (%)	HMF Selectivity (%)	HMF Yield (%)
90	405	95	67	64
95	315	98	69	67
100	225	97	72	70
105	137	98	72	71
110	129	99	71	70
115	105	99	67	66
120	90	99	68	67

**Table 2:** Effect of catalyst particle size on fructose conversion, HMF selectivity and HMF yield. Reaction conditions: 0.5 M fructose, 15 vol % water in HFIP, 22 g/L (23.8 mol %) Lewatit K2420 and 105 °C.

Particle Size (µm)	Reaction Time (min)	Fructose Conversion (%)	HMF Selectivity (%)	HMF Yield (%)
Original (>500)	137	98	72	71
150–300	120	99	76	75
25–100	120	99	77	76

## Supplementary Materials

The supplementary materials are available online. Table S1. Properties of applied dry cationic ion-exchange resins. All data were provided by the manufacturer. Table S2. Chemical and physical characteristics as well as safety parameters of 1,1,1,6,6,6-hexafluoroisopropanol (HFIP).
