# Supplementary material for: Catalytic Dehydration of Fructose to 5-Hydroxymethylfurfural (HMF) in Low-Boiling Solvent Hexafluoroisopropanol (HFIP)
Source: Molecules. 2018 Jul 26;23(8):1866. doi: 10.3390/molecules23081866 (PMC6222439; doi:10.3390/molecules23081866)
Supplement: Supplementary file 1 [file molecules-23-01866-s001.pdf]

molecules
MDPI

Article

Catalytic dehydration of fructose to 5-hydroxymethylfurfural (HMF) in low boiling solvent hexafluoroisopropanol (HFIP)

Sarah Tschirner, Eric Weingart, Linda Teevs and Ulf Prüße*

Thünen Institute of Agricultural Technology, Bundesallee 47, 38116 Braunschweig;
* Correspondence: ulf.pruesse@thuenen.de; Tel.: +49 531 596 4270

Supplementary Materials:

Table S1. Properties of applied dry cationic ion-exchange resins. All data were provided by the manufacturers.

catalyst name	matrix	acid site concentration [mmol/g]	T_{max} [°C]	pore diameter [nm]	BET surface area [m²/g]	pore volume [cm³/g]
Lewatit K2420	styrene/DVB[1] macroporous	5.4	150	53	30	0.4
Lewatit K2431	styrene/DVB[1] macroporous	1.2[2]	130	40	25	0.35
Lewatit K2620	styrene/DVB[1] macroporous	5.2	140	41	33	0.45
Amberlyst 15	styrene/DVB[1] macroporous	4.7	120	30	53	0.4
Amberlyst 16	styrene/DVB[1] macroporous	4.7	130	25	30	0.2
Amberlite IR-120 (H)	styrene/DVB[1] gel	4.5	135	<0.5	<0.1	-

[1] DVB = divinylbenzene. [2] wet form [mmol/L].

Table S2. Chemical and physical characteristics as well as safety parameters of 1,1,1,6,6,6-hexafluoroisopropanol (HFIP).

boiling point [°C]	melting point [°C]	density [g/cm³]	pK_a	GWP (global warming potential)	Classification according to regulation (EC) No 1272/2008
58	-4	1.6	9.3	200	GHS 05, GHS 07, GHS 08
